# Compounds that correct F508del-CFTR trafficking can also correct other protein trafficking diseases: an in vitro study using cell lines

**DOI:** 10.1186/1750-1172-8-11

**Published:** 2013-01-14

**Authors:** Heidi M Sampson, Hung Lam, Pei-Chun Chen, Donglei Zhang, Cristina Mottillo, Myriam Mirza, Karim Qasim, Alvin Shrier, Show-Ling Shyng, John W Hanrahan, David Y Thomas

**Affiliations:** 1 Department of Biochemistry, McGill University, 3655 Promenade Sir William Osler, McIntyre Medical Building, Montréal, Québec H3G 1Y6, Canada; 2 Department of Physiology, McGill University, 3655 Promenade Sir William Osler, McIntyre Medical Building, Montréal, Québec H3G 1Y6, Canada; 3 Department of Biochemistry and Molecular Biology, Oregon Health & Science University, Mail Stop L224, 3181S.W. Sam Jackson Park Road, Portland, OR, 97239-3098, USA

**Keywords:** Protein trafficking, Corrector, Proteostasis, CFTR, hERG, KCNH2, AVPR2, V2R, ABCC8, SUR1

## Abstract

**Background:**

Many genetic diseases are due to defects in protein trafficking where the mutant protein is recognized by the quality control systems, retained in the endoplasmic reticulum (ER), and degraded by the proteasome. In many cases, the mutant protein retains function if it can be trafficked to its proper cellular location. We have identified structurally diverse correctors that restore the trafficking and function of the most common mutation causing cystic fibrosis, F508del-CFTR. Most of these correctors do not act directly as ligands of CFTR, but indirectly on other pathways to promote folding and correction. We hypothesize that these proteostasis regulators may also correct other protein trafficking diseases.

**Methods:**

To test our hypothesis, we used stable cell lines or transient transfection to express 2 well-studied trafficking disease mutations in each of 3 different proteins: the arginine-vasopressin receptor 2 (AVPR2, also known as V2R), the human ether-a-go-go-related gene (KCNH2, also known as hERG), and finally the sulfonylurea receptor 1 (ABCC8, also known as SUR1). We treated cells expressing these mutant proteins with 9 structurally diverse F508del-CFTR correctors that function through different cellular mechanisms and assessed whether correction occurred via immunoblotting and functional assays. Results were deemed significantly different from controls by a one-way ANOVA (*p* < 0.05).

**Results:**

Here we show that F508del-CFTR correctors RDR1, KM60 and KM57 also correct some mutant alleles of other protein trafficking diseases. We also show that one corrector, the cardiac glycoside ouabain, was found to alter the glycosylation of all mutant alleles tested.

**Conclusions:**

Correctors of F508del-CFTR trafficking might have broader applications to other protein trafficking diseases.

## Background

Many protein trafficking diseases are caused by mutations that cause temperature sensitive misfolding and can be partially corrected by incubation at low temperature (< 30°C) or treatment with high concentrations of osmolytes such as glycerol, trimethylamine oxide or 4-phenylbutyrate [1-3]. However, most of these are not feasible therapeutic strategies. Small molecule correctors have recently been identified for several protein trafficking diseases including cystic fibrosis (CF) [4-12]. Some of these correctors function as pharmacological chaperones and bind directly to the mutant protein to improve its folding [10,13]. Pharmacological chaperones for CF are predicted to be specific for the cystic fibrosis transmembrane conductance regulator (CFTR) and not correct other mutant proteins unless they share structural similarity with CFTR. Other correctors target cellular pathways and act through proteostasis [14-19]. These proteostasis regulators are predicted to correct diverse protein trafficking diseases. The F508del-CFTR correctors chosen for this study include several structurally distinct compounds that act on different cellular pathways. They include the phosphodiesterase inhibitors KM57 and KM60 [18], poly ADP-ribose polymerase (PARP) inhibitors ABT-888 [14,20] and latonduine [15], the cardiac glycoside ouabain [19], the cyclooxygenase inhibitor glafenine [17], carbamazepine, a sodium channel blocker which promotes proteasomal and autophagic degradation of mutant proteins [21,22], and RDR1, a corrector that we originally reported to act as a pharmacological chaperone [12], but whose mechanism of action remains unclear [23]. We also included the F508del-CFTR pharmacological chaperone VRT-325 [10,13]. Notably, VRT-325 has been reported to correct other mutated proteins besides CFTR [10].

We investigated mutants from a diverse set of well-studied protein trafficking diseases including the nephrogenic diabetes insipidus mutations V206D and L292P in the arginine-vasopressin receptor 2 (AVPR2, also known as V2R) [24,25], the LQTS2 mutations G601S and F805C in the human ether-a-go-go-related gene (KCNH2, also known as hERG) [26,27], and finally the persistent hyperinsulinemic hypoglycemia of infancy (PHHI, also known as congenital hyperinsulinism) mutations A116P and V187D in the sulfonylurea receptor 1 (ABCC8, also known as SUR1) [10,28,29], a component of the K_ATP_ channel. All these proteins are localized to the plasma membrane and have one or more glycosylation sites that can be used to monitor their progression through the secretory pathway by immunoblotting, where ER-retained (core-glycosylated) proteins migrate faster than rescued (complex-glycosylated) proteins. Additionally, there is currently no treatment for these diseases that addresses the basic protein trafficking defect.

We show that RDR1 rescues a mutant of the only other ATPase-binding cassette (ABC) protein related to CFTR in our study, SUR1. Additionally, we show that the phosphodiesterase inhibitors KM60 and KM57 rescue the trafficking of a hERG mutant. Finally, we show that ouabain modifies the glycosylation of all mutants tested, albeit without correcting the trafficking of proteins other than CFTR. Our results provide impetus for testing F508del-CFTR correctors on other trafficking disease mutants, which could generate new leads with therapeutic benefit to several rare diseases.

## Methods

### Cell lines and constructs

The GFP-tagged V2R WT clone was generated by site-directed mutagenesis of plasmid V2R-V206D.GFP, kindly provided by Dr. Peter Deen (NCMLS, The Netherlands), using the primers 5^′^-CCGTCGCACCTATGTTACCTGGATTGCCC-3^′^ and 5^′^-GGGCAATCCAGGTAACATAGGTGCGACGG-3^′^. HA-FLAG-tagged V2R WT and HA-tagged V2R L292P in pcDps, kindly provided by Dr. Torsten Schöneberg (University of Leipzig, Germany), were sub-cloned into pcDNA3.1 using *Bgl*II and *Spe*I sites in pcDps and *Bam*HI and *Xba*I sites in pcDNA3.1 (WT) or *Bgl*II and *Xho*I sites (L292P). HEK cells stably expressing HA-tagged hERG G601S [30] were kindly provided by Dr. Eckhard Ficker (Case Western Reserve University, U.S.A.). HA-tagged hERG F805C mutant and WT plasmids have been described previously [31]. FLAG-tagged hamster SUR1 WT, A116P and V187D mutants and rat Kir6.2 plasmids have been described previously [32].

### Transfection and compound treatment

HeLa cells were seeded at a density of 8.0x10^6 cells/flask and transiently transfected with 18 μg of plasmid and 63 μl of Fugene HD overnight. The following day, the cells were trypsinized and seeded into 6-well plates at 1.0-1.5x10^6 cells/well. The following day, the cells were treated with compounds or vehicle at a final DMSO concentration of 0.1% for 24 h. HEK cells stably expressing 3HA-hERG G601S were seeded in 6-well dishes as above and treated with compounds for 48 h.

### Immunoblotting

Cells were harvested in RIPA buffer [150 mM NaCl, 20 mM Tris, 0.01% SDS, 0.08% sodium deoxycholate, 1% Triton-X-100, pH 8.0 with protease inhibitors (Roche)]. Equal amounts of protein were separated by SDS-PAGE and transferred onto nitrocellulose. Blots were blocked in PBST (137 mM NaCl, 2.68 mM KCl, 9.31 mM Na_2_HPO_4_, 2.45 mM KH_2_PO_4_, pH 7.4, containing 0.1% Tween-20) containing 5% skim milk and probed with appropriate primary antibodies (rabbit anti-hERG, Calbiochem; SUR1: mouse anti-FLAG M2, Sigma; V2R V206D: mouse anti-GFP, Roche; V2R L292P: mouse anti-HA, Covance). The blots were washed in PBST and probed with HRP-conjugated secondary antibodies. Chemiluminescent substrates were added and the blots were exposed to film.

### Densitometry

The relative levels of each HERG glycoform for selected treatments were estimated by densitometry using Photoshop (Adobe Inc.). The values reported are expressed as means +/− SD (n = 3).

### Patch clamping (hERG G601S)

Whole-cell currents were recorded from HEK hERG G601S cells after 48 h of treatment with compounds. An Alembic VE-2 patch clamp amplifier (Alembic Instruments Inc., Montreal) that provides full series resistance compensation was used to record whole-cell currents. The bath solution contained 137 mM NaCl, 4 mM KCl, 1.8 mM CaCl_2_, 1 mM MgCl_2_, 10 mM glucose and 10 mM HEPES (pH 7.4). Patch pipettes were made from borosilicate glass with a resistance of 3–6 megaohms. Intracellular pipette solution contained 130 mM KCl, 1 mM MgCl_2_, 5 mM EGTA, 5 mM MgATP and 10 mM HEPES (pH 7.2). Cells were held at a potential of −80 mV. hERG tail currents were evoked by 4 s steps ranging from −60 to +50 mV in increments of 10 mV, with each step followed by a repolarizing step to -50 mV for 2 s. Data were collected and analyzed with Clampex 8 and Prism software.

### Rubidium efflux (SUR1 mutants)

COSm6 cells were plated onto 6-well plates and transiently transfected with 0.6 μg SUR1 and 0.4 μg Kir6.2 plasmids and 3 μl Fugene overnight. The following day, the cells were treated with compounds or vehicle for 24 h. Cells were incubated for an additional 12 h in culture medium containing ^86^RbCl (1 μCi/ml); during this period, compounds were present in the first 10 h followed by a 2-h washout. Before measurement of ^86^Rb^+^ efflux, cells were incubated for 30 min at room temperature in Krebs-Ringer solution (118 mM NaCl, 2.5 mM CaCl_2_.H_2_O, 1.2 mM KH_2_PO_4_, 4.7 mM KCl, 25 mM NaHCO_3_, 1.2 mM MgSO_4_, 10 mM HEPES, pH 7.4) with metabolic inhibitors (2.5 μM oligomycin and 1 mM 2-deoxy-D-glucose) to open the channel. At selected time points the solution in the well was collected and fresh solution added. At the end of a 40-min period, the cells were lysed. The ^86^Rb^+^ in the collected solution and the cell lysate was counted. The percentage efflux at each time point was calculated as the cumulative counts in the collected solution divided by the total counts from the solutions and the cell lysate.

### Cyclic AMP assays

Cyclic AMP assays (Sigma-Aldrich) were performed according to the manufacturer’s directions. Briefly, cells transfected with V2R plasmids were seeded at 3.0x10^5 cells/well in 24-well dishes and treated the following day for 24 h with compound, in quadruplicate. The following day, the cells were washed with warm PBS and treated with 250 μM IBMX for 10 min to inhibit any phosphodiesterase activity [33]. The medium was aspirated and replaced with either fresh medium containing 0.1% DMSO (unstimulated control, in duplicate) or fresh medium containing 100 nM DDAVP (stimulated, in duplicate) for 10 min to stimulate V2R at the cell surface [33]. Cells were washed with PBS and lysed in 110 μl of 100 mM HCl at room temperature. Supernatants were collected and centrifuged. 50 μl of the supernatant were used in the assay.

### Statistical analyses

Statistical analysis was performed as described in the Figure legends. *P*-values < 0.05 were considered significant.

## Results and discussion

**Figure 1:**
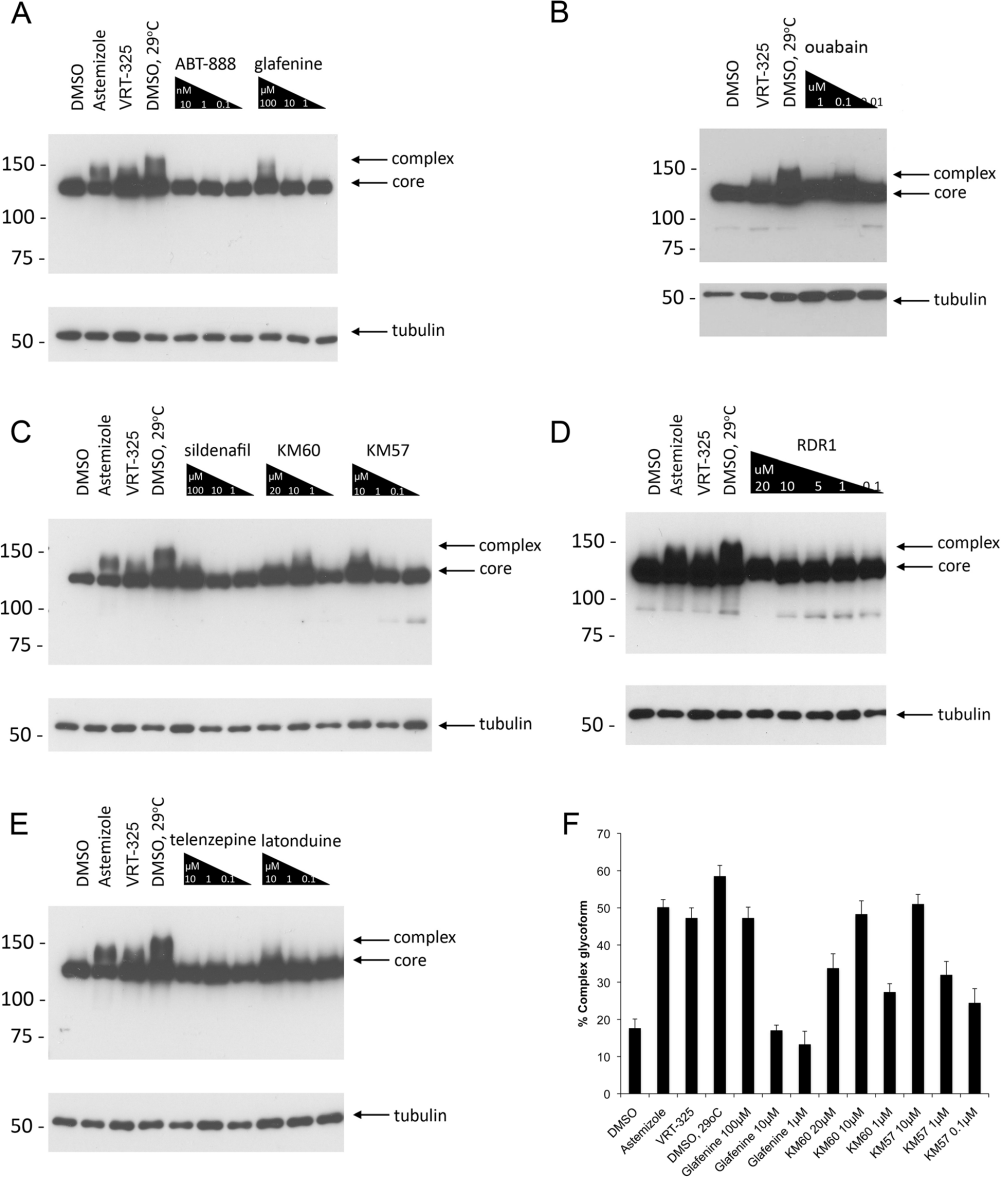
**Some F508del**-**CFTR correctors correct the trafficking of hERG G601S.** Representative immunoblots are shown for decreasing concentrations of ABT-888 and glafenine (**A**), ouabain (**B**), sildenafil, KM60, and KM57 (**C**), RDR1 (**D**), telenzepine and latonduine (**E**). In all blots, negative control (DMSO) and positive controls (astemizole; VRT-325; DMSO, 29°C) are shown. Arrows indicating core-glycosylated hERG G601S, and complex-glycosylated hERG G601S, which represents the corrected protein, are shown. Tubulin is shown as a loading control. (**F**) Densitometric quantitation of core and complex-glycosylated bands for selected treatments is shown. Error bars indicate SD (n = 3).

**Figure 2:**
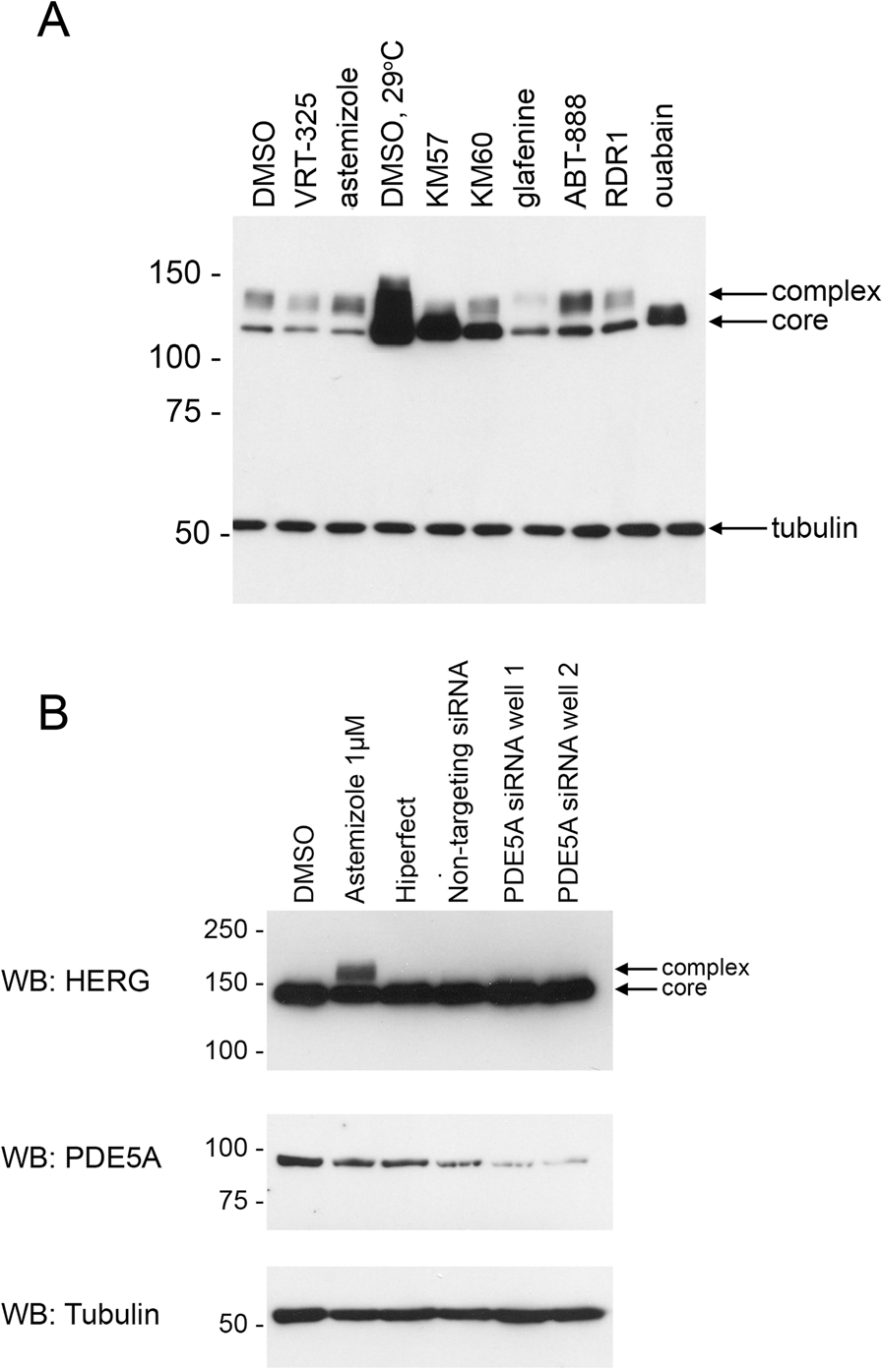
**F508del**-**CFTR trafficking correctors have mixed effects on the trafficking of wild type hERG.** (**A**) Representative blot showing wt hERG treated with selected trafficking correctors as labeled above the immunoblot. Arrows indicate core and complex-glycosylated hERG. Tubulin serves as a loading control. (**B**) siRNA knockdown of PDE5A does not correct the trafficking of hERG G601S. ***Top****: *hERG G601S cells treated with vehicle (DMSO), 1 μM astemizole as a positive control, siRNA transfection reagent alone (HiPerfect), non-targeting siRNA, or two separate wells transfected with 25 nM siRNA against PDE5A. Arrows indicate core and complex-glycosylated hERG. ***Middle****: *PDE5A expression levels in each of the samples described. ***Bottom****: *Tubulin serves as a loading control.

**Figure 3:**
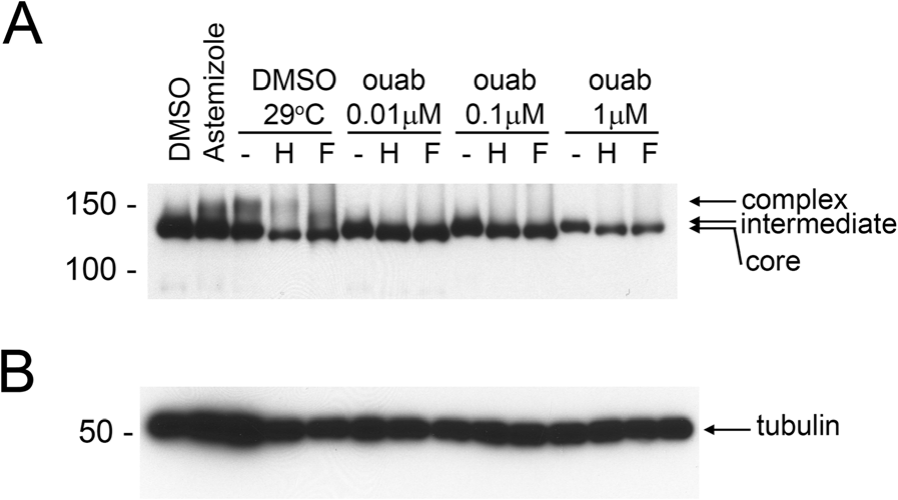
**Ouabain treatment induces an intermediate hERG G601S glycosylation state. **(**A**) Representative blot showing hERG G601S treated with increasing concentrations of ouabain in the absence (−) and presence of endoglycosidase enzymes Endo H (H), PNGase F (F), as indicated. Treatment with vehicle (DMSO) and astemizole are shown as negative and positive controls for correction. Core, intermediate, and complex-glycosylated hERG are marked by arrows. (**B**) Tubulin is shown as a loading control.

**Figure 4:**
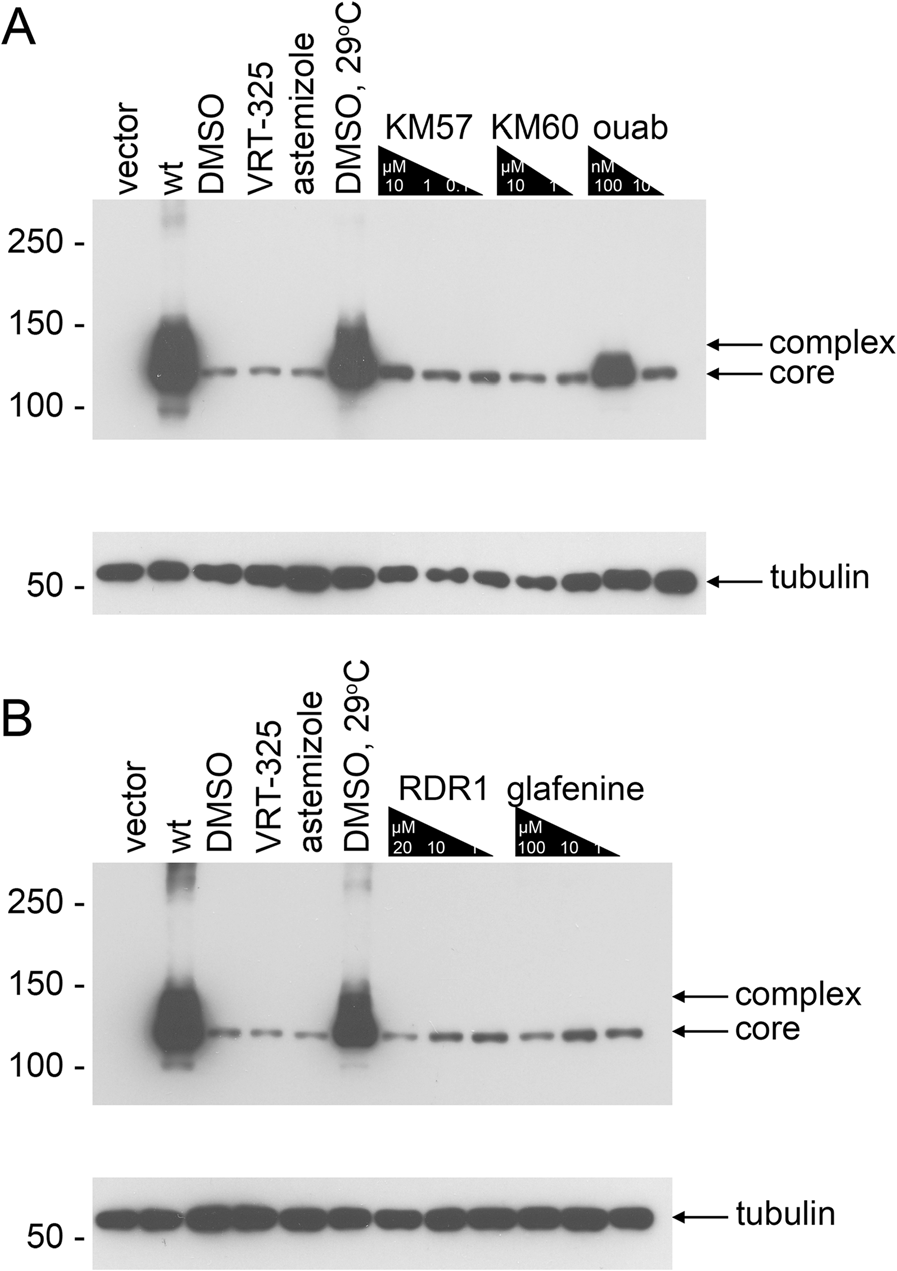
**Few F508del**-**CFTR correctors correct the trafficking of hERG F805C. **(**A**) A representative immunoblot is shown for cells transfected with vector control (vector), wt hERG (wt), and hERG F805C treated with negative control (DMSO), positive control (DMSO 29°C), astemizole, VRT-325, decreasing concentrations of KM60, KM57, and ouabain (ouab). Arrows indicate core and complex-glycosylated hERG F805C. Tubulin is shown as a loading control. (**B**) A representative immunoblot is shown for cells transfected with vector control (vector), wt hERG (wt), and hERG F805C treated with negative control (DMSO), positive control (DMSO 29°C), astemizole, VRT-325, decreasing concentrations of RDR1 and glafenine. Arrows indicate core and complex-glycosylated hERG F805C. Tubulin is shown as a loading control.

**Figure 5:**
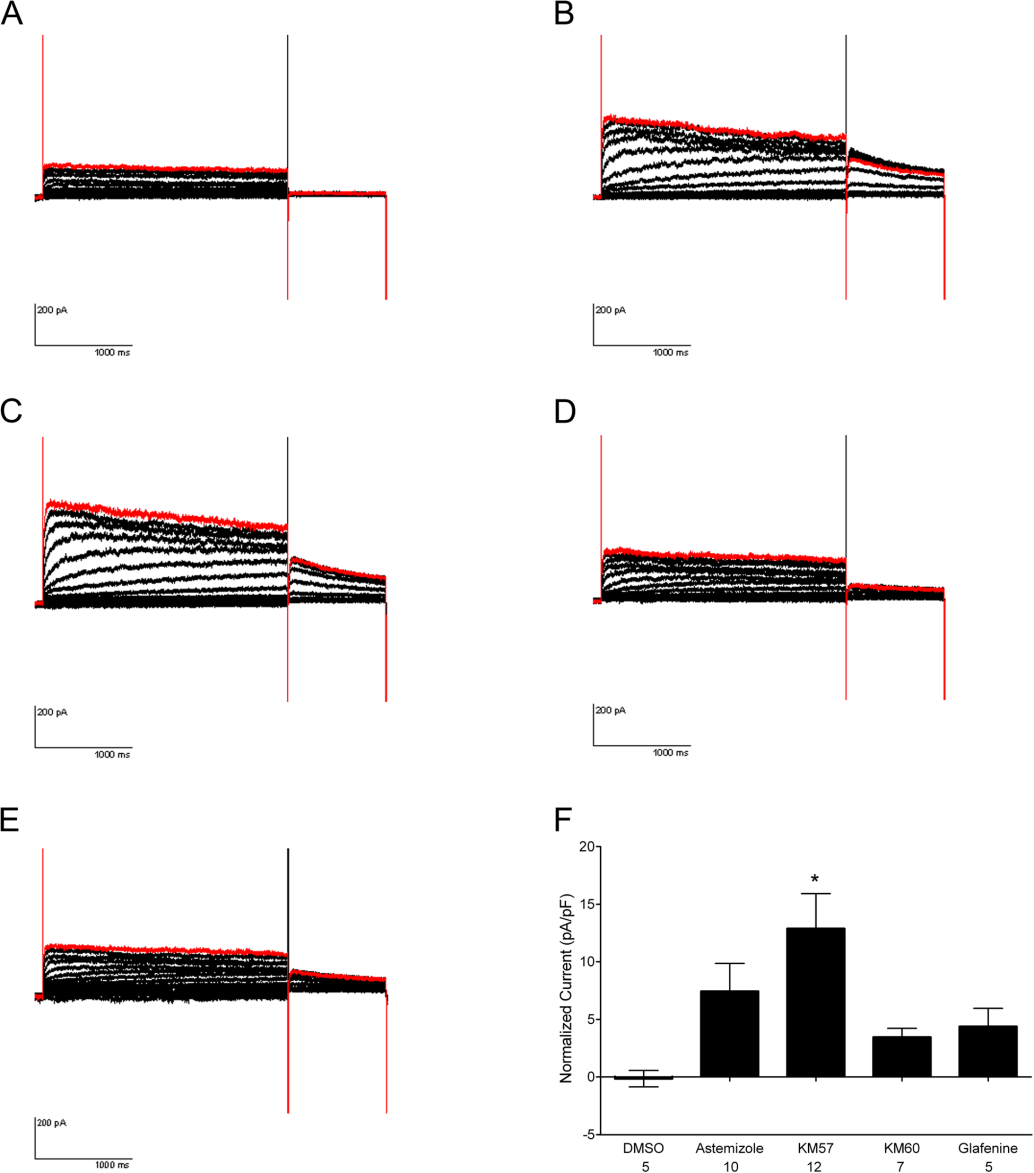
**F508del**-**CFTR correctors result in functional hERG G601S channels.** Representative patch clamping traces are shown for vehicle-treated cells (**A**), astemizole treatment and washout (**B**), KM57 treatment and washout (**C**), KM60 treatment and washout (**D**), and glafenine treatment and washout (**E**). Quantitative analysis of the tail currents is shown in (**F**). Only KM57 treatment resulted in a significant increase in tail currents using one-way ANOVA and Bonferroni’s multiple comparison test (*p *=0.0131). Numbers below the treatments indicate n.

We tested whether F508del-CFTR correctors could restore hERG G601S trafficking and function. As shown in Figure 1, several correctors including KM57, KM60, glafenine, ouabain, and latonduine modified the glycosylation of this hERG mutant as judged by the appearance of slower-migrating, complex-glycosylated forms of the protein, indicating partial rescue. Treatment with glafenine, KM60 and KM57 resulted in the most prominent complex-glycosylated forms of HERG G601S, while latonduine treatment appeared to yield slightly less rescue. Low temperature, VRT-325 and the hERG channel blocker astemizole served as positive controls [10,26]. Interestingly, low temperature treatment produced an additional slower migrating species that was not present in the other positive controls (Figure 1A-E). Treatment with the cardiac glycoside ouabain, a known inhibitor of trafficking of the wild type hERG channel [34] (see also Figure 2A), produced an intermediate glycoform (Figure 1B, Figure 3). Densitometric quantitation of some of the treatments is shown in Figure 1F. We also tested these correctors on the trafficking of the hERG F805C mutant. Unlike hERG G601S, the F805C mutant was only corrected by low temperature and an intermediate glycoform was produced with ouabain treatment (Figure 4). Given that sildenafil, KM60 and KM57 are all phosphodiesterase inhibitors, and KM60 and KM57 were more potent correctors of hERG G601S, we chose to pursue only KM60 and KM57 in our study. To determine if correction resulted in a functional hERG G601S polypeptide, we used patch clamp recording (Figure 5). A short (1 hr) washout of the correctors was necessary to observe an increase in hERG tail currents (Figure 5). Exposure and washout of KM57 (Figure 5C), KM60 (Figure 5D), glafenine (Figure 5E), and astemizole (Figure 5B) all resulted in increased hERG G601S tail currents (Figure 5F). DMSO vehicle (0.1%) served as a negative control (Figure 5A). Treatment and washout of KM57 resulted in a statistically significant increase in hERG G601S tail currents above the DMSO control (Figure 5F). Since a washout step was necessary to observe tail currents, the mechanism used by these correctors may be through direct binding to, and inhibition of, the mutant channel [35]. Alternatively, KM57 and KM60 may be acting through a proteostasis regulator. To test this hypothesis, we used siRNA knockdown of PDE5A, the purported target of KM60 and KM57. Interestingly, knockdown of PDE5A did not result in an increase in hERG G601S trafficking (Figure 2B). Additionally, treatment of wild type hERG with these correctors reduced the amount of complex-glycosylated mature hERG detected by immunoblotting (Figure 2A). In contrast, low temperature treatment of wild type hERG induced significantly greater amounts of both the core and complex-glycosylated forms of the protein, as would be expected due to the more favorable conditions for protein folding [19]. These results suggest that KM57 and KM60 may be acting through a proteostasis regulator other than PDE5A to affect hERG trafficking.

**Figure 6:**
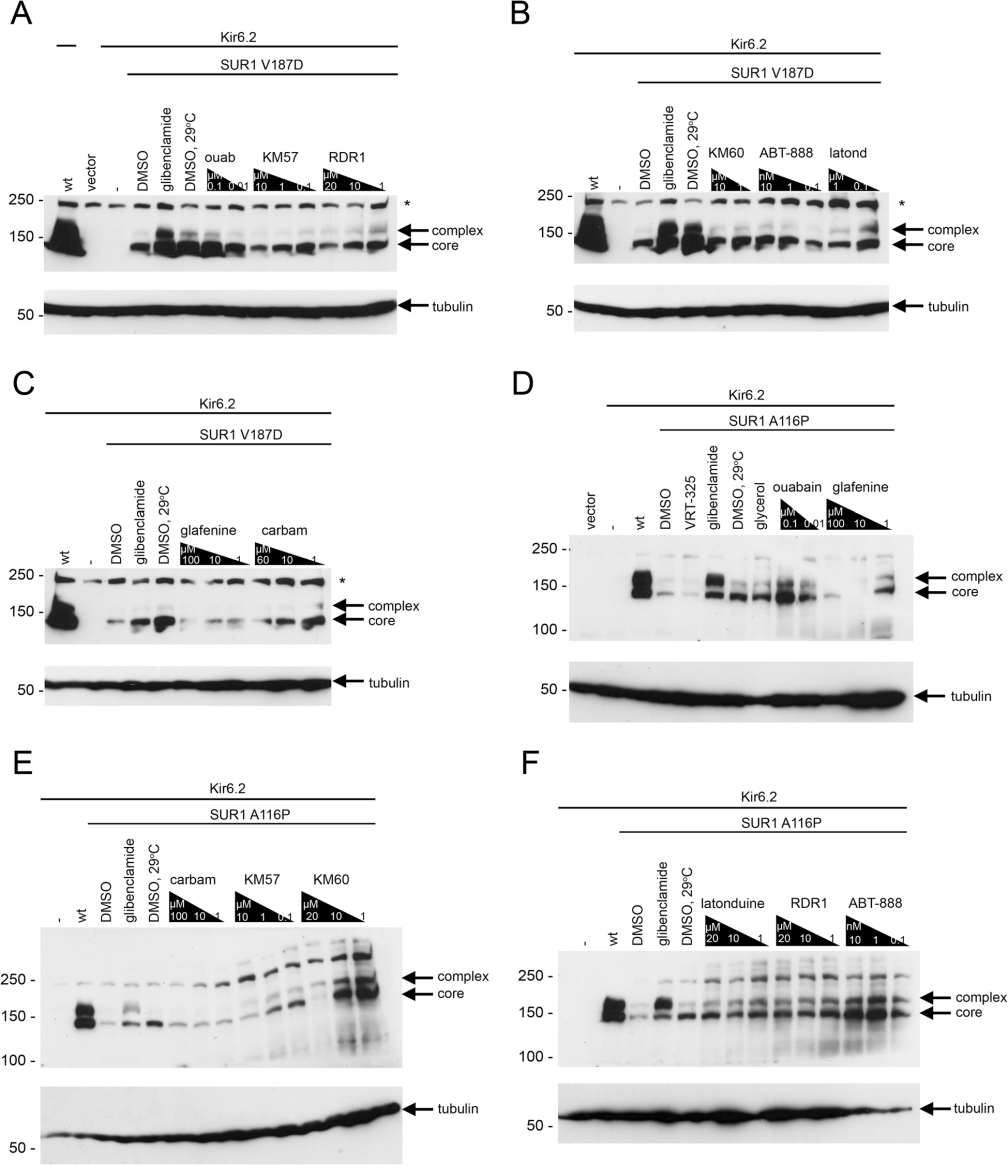
**F508del**-**CFTR correctors correct the trafficking of SUR1 mutants V187D and A116P. **Representative immunoblots are shown for SUR1 V187D (**A-C**) and SUR1 A116P (**D-F**). Representative immunoblot for cells expressing SUR1 V187D treated with decreasing concentrations of ouabain (ouab), KM57, and RDR1 (**A**), KM60, ABT-888, and latonduine (latond) (**B**), glafenine and carbamazepine (carbam) (**C**). Representative immunoblot for cells expressing SUR1 A116P treated with 10% glycerol (glycerol), decreasing concentrations of ouabain and glafenine (**D**), carbamazepine (carbam), KM57, and KM60 (**E**), latonduine, RDR1, ABT-888 (**F**). In all blots, lanes expressing vector controls (vector), Kir6.2 in the absence of SUR1 (−), and wild type SUR1 (wt) are shown. Lanes where Kir6.2 and SUR1 V187D or SUR1 A116P are expressed are indicated by a line above the corresponding lanes. Each blot has a vehicle control (DMSO) and positive controls (glibenclamide; DMSO, 29°C). Arrows indicate core-glycosylated SUR1 protein (core), complex-glycosylated SUR1 protein (complex, indicating rescue). Tubulin is shown as a loading control. In panels A-C, an asterisk indicates a non-specific band.

**Figure 7:**
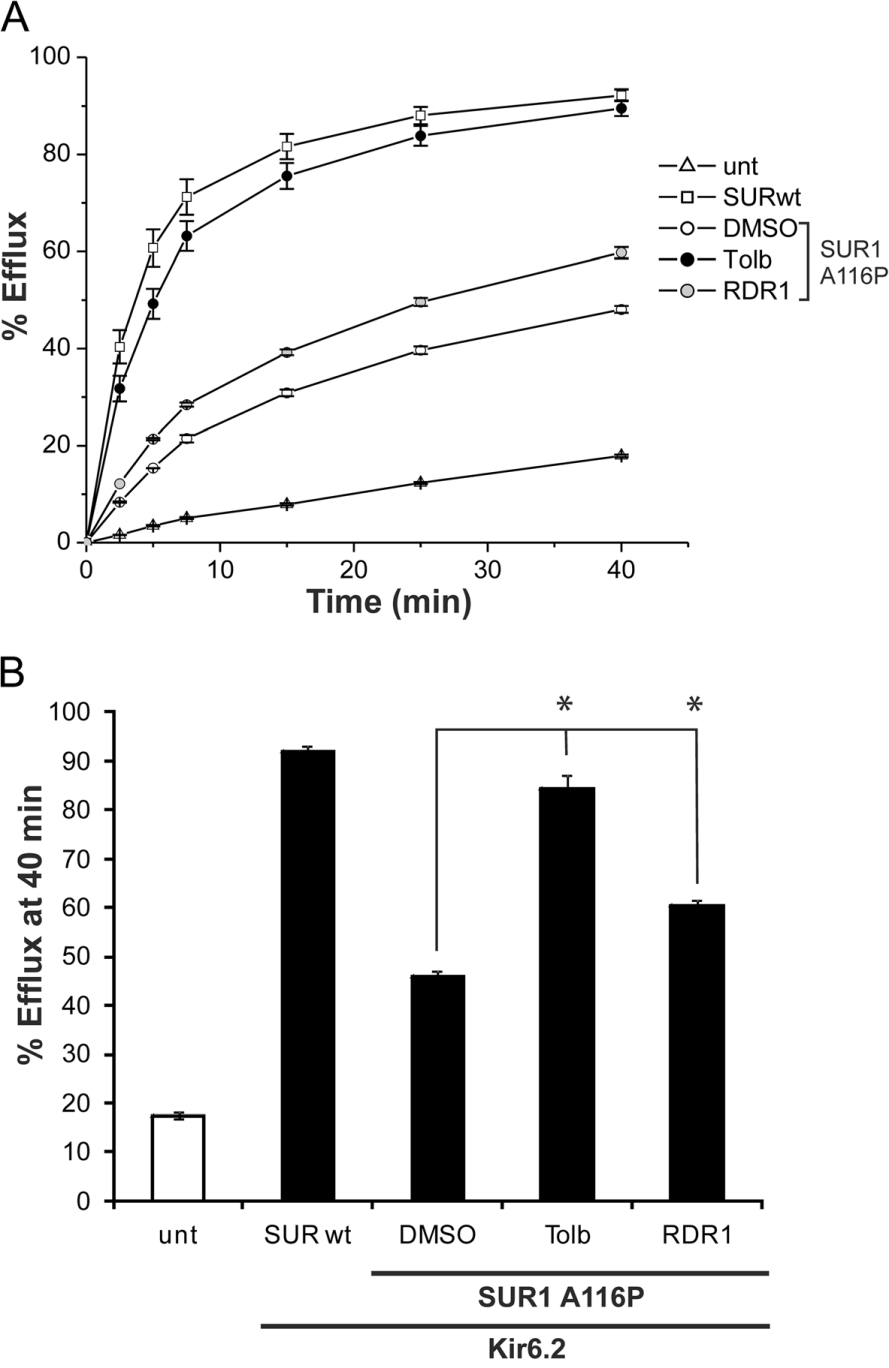
**The F508del-CFTR corrector RDR1 improves the function of the SUR1 A116P mutant. **(**A**) Representative ^86^Rb^+^ efflux profiles from COS cells transiently transfected with SUR1 A116P and wt Kir6.2 and treated with 0.1% DMSO, 300μM tolbutamide (a reversible sulfonylurea) or 10μM RDR1 as described in the **METHODS**. Non-transfected cells (unt) and cells transiently transfected with wt SUR1 and Kir6.2 (SURwt) served as controls. Each data point is the average of triplicates performed in the same experiment with the exception of RDR1-treated group (RDR1) in which each data point is the average of six samples. Error bars indicate S.E.M. (**B**) Comparison of % ^86^Rb^+ ^efflux at the 40-min time point in cells treated with different compounds. Only RDR1 resulted in significant improvement of channel activity. Each data point is the mean ± S.E.M. (n = 7) from 4 independent experiments; *, p < 0.05 compared to DMSO treated group by one-way ANOVA and Dunnett’s *post-hoc *test.

We next tested the SUR1 mutants V187D and A116P for correction with F508del-CFTR correctors. Treatment with several correctors yielded the complex-glycosylated form of these mutants, indicating partial rescue (Figure 6). To determine whether correction of the SUR1 mutants leads to functional recovery of K_ATP_ channels, we performed ^86^Rb^+^ efflux assays to assess channel activity in intact cells in response to metabolic inhibition (Figure 7). In contrast to cells expressing wild type SUR1 protein, those expressing the SUR1 A116P mutant showed only low levels of channel activity due to loss of surface expression after treatment with the DMSO control. Exposing A116P-expressing cells to the reversible sulfonylurea drug tolbutamide for > 24 h followed by a washout 2 h prior to the assay led to almost complete recovery of channel activity, as reported previously [32]. Among the correctors that enhanced the processing of A116P, KM57, KM60 and RDR1 were tested for their effects on functional recovery of the mutant channel. Only RDR1 improved channel function significantly (Figure 7 and data not shown). We previously reported RDR1 as a pharmacological chaperone for F508del-CFTR that binds to and stabilizes the first nucleotide-binding domain (NBD1) against thermal denaturation [12]. However, additional studies since then suggest that RDR1 does not exert its effect through NBD1 [23], although RDR1 may act still as a pharmacological chaperone for F508del-CFTR by affecting other regions of the protein. Interestingly, sulfonylureas correct the trafficking of A116P and V187D mutants by binding to sites outside the affected domain in SUR1, and possibly also with a weak affinity site in Kir6.2 [36]. The mechanism of correction employed by RDR1 may be similar to that proposed for sulfonylureas, perhaps mediated by binding at a different site in SUR1. Alternatively, RDR1 may be acting as a proteostasis regulator through an unidentified protein that exerts its effects on both SUR1 and CFTR.

**Figure 8:**
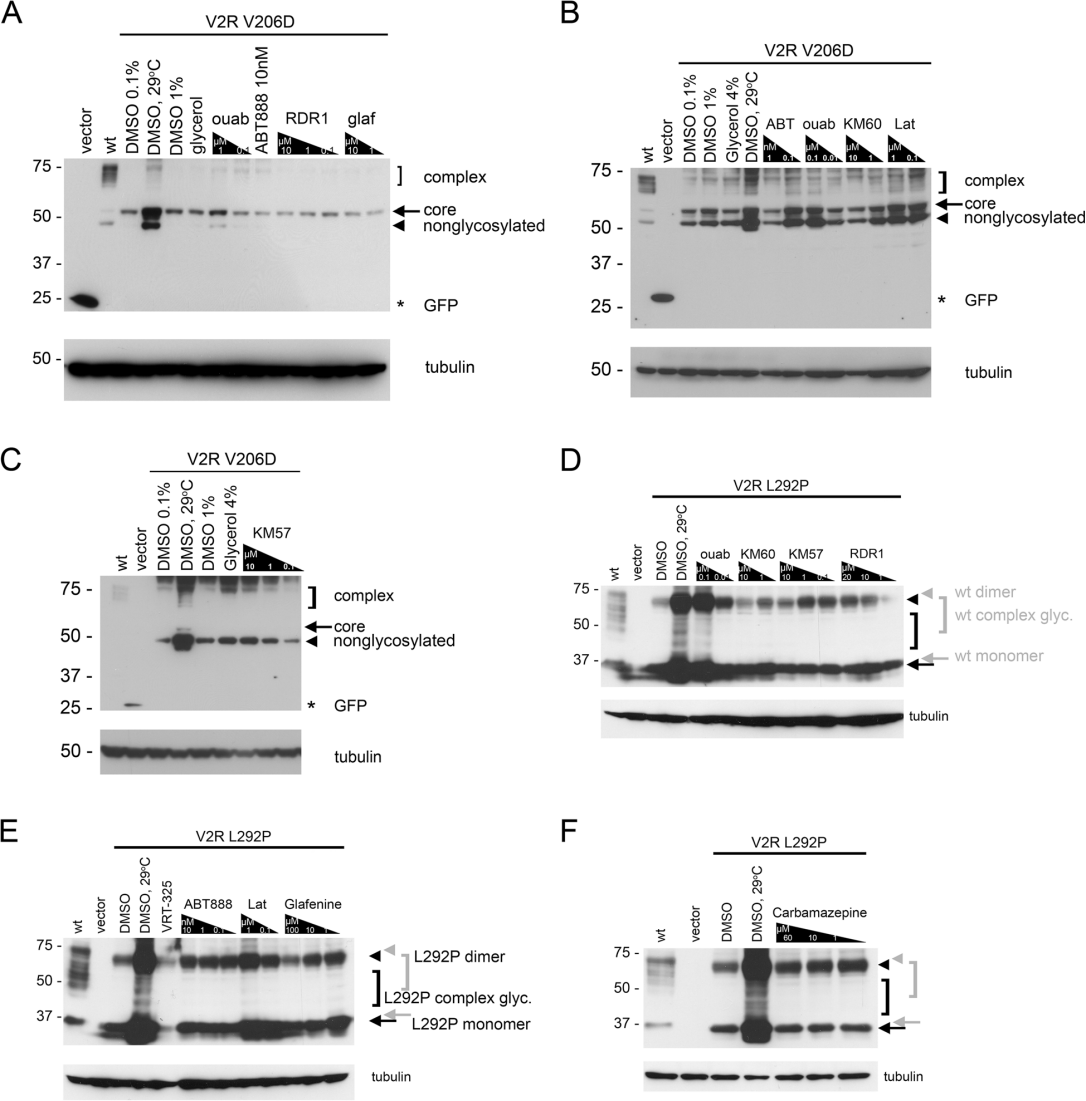
**V2R mutants show limited correction upon F508del-CFTR corrector treatment.** Representative blots are shown for V2R V206D (**A-C**) and V2R L292P (**D-F**). Representative immunoblot for cells expressing V2R V206D treated with decreasing concentrations of ouabain (ouab), 10nM ABT-888, decreasing concentrations of RDR1 (RDR1), and glafenine (glaf) (**A**); ABT-888 (ABT), ouabain (ouab), KM60, and latonduine (Lat) (**B**); KM57 (**C**). Representative immunoblot for cells expressing V2R L292P treated with decreasing concentrations of ouabain (ouab), KM60, KM57, RDR1 (**D**); ABT-888 (ABT888), latonduine (Lat), and glafenine (**E**); carbamazepine (**F**). In all blots, lanes expressing vector controls (vector), and wild type V2R (wt) are shown. A line above the corresponding lanes indicates where V2R V206D or V2R L292P is expressed. Each blot has a vehicle control (DMSO or DMSO 0.1%) and a positive control (DMSO, 29°C). For V2R V206D, two other positive controls are also included (1% DMSO, glycerol). In (**A-C**), an arrow indicates core glycosylated V2R protein (core), a bracket indicates complex-glycosylated V2R protein (complex, indicating rescue), an arrowhead indicates nonglycosylated protein (nonglycosylated), and a star indicates GFP. In (**D-F**), a black arrow indicates mutant core glycosylated monomer, a black bracket indicates mutant complex-glycosylated monomer, and a black arrowhead indicates mutant dimer. The same symbols are also shown in gray to indicate the wild type V2R protein, which has a slightly slower mobility on SDS-PAGE. In all blots tubulin is shown as a loading control.

**Figure 9:**
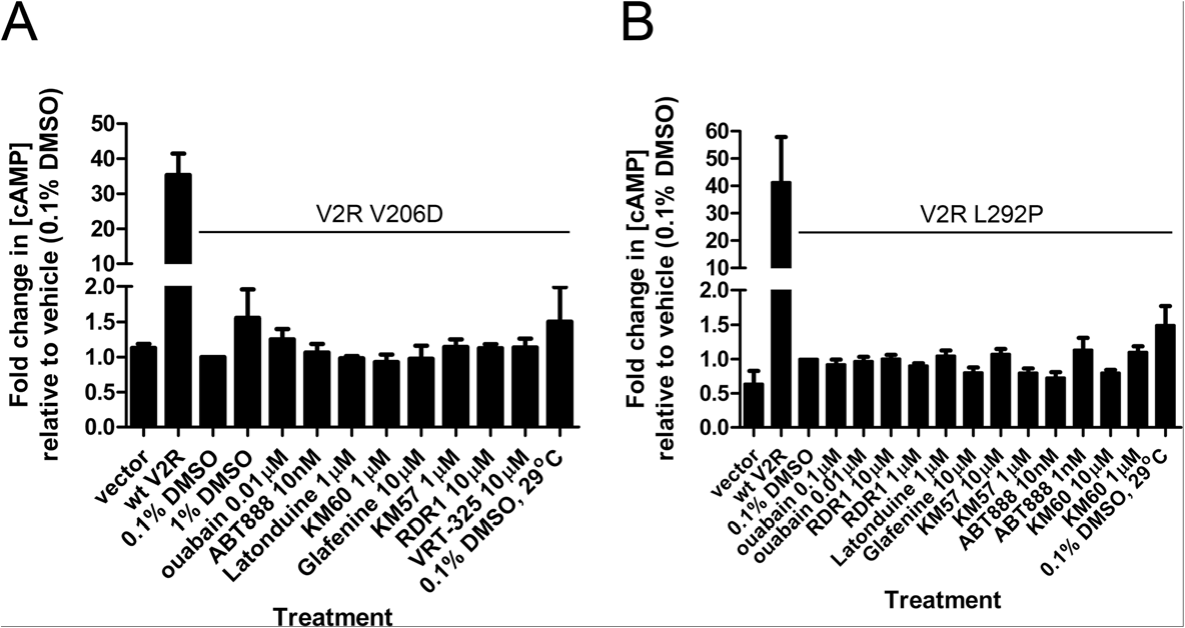
**F508del-CFTR correctors do not functionally correct V2R mutants. **Cyclic AMP accumulation assays are shown for cells expressing V2R V206D (**A**) or V2R L292P (**B**) treated with correctors for 24 h. Data shown are means ± S.E.M. (n = 3 independent experiments). None of the compound treatments were statistically significant from the DMSO control with a one-way ANOVA.

We also tested these correctors on the V2R mutants L292P and V206D (Figures 8 and 9). Although several correctors increased the trafficking of V2R V206D from ER to the Golgi compartment as evidenced by the appearance of complex-glycosylated forms of the protein (Figure 8A-C), none of them corrected V2R function following stimulation with a vasopressin analog (DDAVP) (Figure 9). The positive controls (1% DMSO and 29°C) increased cAMP production above the vehicle control with the V2R V206D mutant; however, this difference was not statistically significant (Figure 9A). Cells expressing V2R L292P protein were slightly less amenable to correction by F508del-CFTR correctors than the V206D allele (Figure 8D-F). None of the correctors yielded functional correction upon stimulation (Figure 9B); however, incubation at 29°C resulted in approximately 2-fold more cAMP than at 37°C (Figure 9B). This increase was not statistically significant. These results contrast with previously published results using COS cells where low temperature preincubation increased V2R function significantly [25]. The lack of functional correction may be attributed to the use of different cell types, or to the mutant proteins not reaching the cell surface. This latter possibility seems less likely as recent work by Robben *et al.* shows that intracellular V2R can induce signaling in response to DDAVP stimulation [37].

The results of immunoblotting studies are summarized in Table 1. Only ouabain treatment altered the glycosylation of all mutants. RDR1 correction was the most protein-specific as it only corrected the trafficking of SUR1, the protein structurally related to CFTR in our test set [38]. However, the other correctors did appear to be partially allele-specific. They corrected at least one protein besides F508del-CFTR, but not necessarily both mutant alleles of the same protein.

**Table 1:** F508del-CFTR corrector compounds show distinct profiles of correction for other ER-retained proteins

**Corrector**	**CFTR F508del**	**hERG G601S**	**hERG F805C**	**SUR1 A116P**	**SUR1 V187D**	**V2R L292P**	**V2R V206D**
VRT-325	+	+	-	-	ND	-	ND
Glycerol	+	ND	ND	+	ND	ND	+/−
29°C	++	+	+	+	+	++	++
KM60	+	+	-	+	+	-	+
KM57	+/−	++	-	+/−	-	-	+/−
ABT-888	+	-	ND	+	+	-	+
Glafenine	+	+	-	+	-	-	-
RDR1	+	-	-	+	+	-	-
Ouabain	+	+/−*	+*	+	+	+	+
Carbamazepine	+	-	ND	-	+/−	-	ND
Latonduine	+	+/−	-	+	++	+/−	+
Astemizole	ND	+	-	ND	ND	ND	ND
Glibenclamide	ND	ND	ND	++	++	ND	ND

A qualitative assessment of correction as determined by glycosylation status in immunoblotting is shown for each mutation following treatment with a corrector compound, where “-“ indicates no correction observed, “+/−“ indicates slight correction, “+” and “++” indicate more and best correction observed, respectively, and “ND” indicates not determined. Asterisks indicate that an intermediate glycoform was observed, but no correction. Astemizole and glibenclamide were included as positive controls for correction of hERG G601S and SUR1 mutants, respectively.

Among the 9 correctors tested, only the cardiac glycoside ouabain altered the glycosylation of all mutant alleles for these three different ER-retained proteins. The only condition that corrects all mutant alleles is incubation at low temperature. Interestingly, when the transcriptional signatures of ouabain and low temperature are compared, they show significant overlap [19], which may account for the altered glycosylation state of ER-retained mutant proteins. Although the exact mechanism employed by ouabain to elicit trafficking correction of F508del-CFTR has not been elucidated, it is known to inhibit the Na+/K + ATPase [39]. The inhibition of Na+/K + ATPase by ouabain leads to intracellular calcium fluctuations and altered glycosylation [40], which, together, could allow ER-retained proteins to escape ER-related protein quality control mechanisms. Except for F508del-CFTR, ouabain does not lead to functional correction of the two V2R trafficking mutants that we tested. This discrepancy might be due to the proteins escaping the ER, but not reaching the cell surface where they function. Alternatively, ouabain may not elicit enough correction for a functional response to be detected; suggesting that further optimization of its chemical scaffold or of the optimal dose would be necessary.

## Conclusions

Taken together, the results provide a compelling case for testing novel F508del-CFTR correctors on mutated proteins in other trafficking diseases. Utilizing the hits identified in high throughput screens for correctors of F508del-CFTR can accelerate the development of correctors for the protein trafficking defect underlying other genetic diseases. A deeper understanding of the targets and mechanisms of action of proteostasis regulators will also be important in the development of these therapeutics.

## Competing interests

The authors declare no competing financial interests.

## Authors’ contributions

HMS, HL, PC, AS, S-LS, and DYT designed the experiments. HMS performed the immunoblotting and cAMP assays. HL performed the hERG patch clamping. PC performed the SUR1 rubidium efflux assays. DZ performed the hERG glycosylation enzyme assays. CM, MM and KQ performed critical cloning steps and immunoblotting experiments for V2R HMS, HL, PC, DZ, S-LS, AS JWH and DYT analyzed the data. AS, S-LS, JWH and DYT directed the project. HMS wrote the manuscript with contributions by PC, S-LS, AS, JWH and DYT All authors read and approved the final manuscript.
